# Detrimental effects of tropisetron on permanent ischemic stroke in the rat

**DOI:** 10.1186/1471-2202-9-19

**Published:** 2008-02-06

**Authors:** Eduardo Candelario-Jalil, Eduardo Muñoz, Bernd L Fiebich

**Affiliations:** 1 Neurochemistry Research Group, Department of Psychiatry, University of Freiburg Medical School, Hauptstr. 5, D-79104 Freiburg, Germany; 2 Department of Neurology, University of New Mexico Health Sciences Center, Albuquerque, NM 87131, USA; 3 Departamento de Biología Celular, Fisiología e Inmunología. Universidad de Córdoba, Avenida Menéndez Pidal s/n. 14004, Córdoba, Spain; 4 VivaCell Biotechnology GmbH, Ferdinand-Porsche-Str. 5, D-79211 Denzlingen, Germany

## Abstract

**Background:**

Recent *in vitro *evidence indicates that blockade of 5-hydroxytryptamine (5-HT) receptor 3 (5-HT_3_) is able to confer protection in different models of neuronal injury. The purpose of the present study was to investigate the effect of tropisetron, a 5-HT_3 _receptor antagonist, on infarct size and neurological score in a model of ischemic stroke induced by permanent middle cerebral artery occlusion (pMCAO) in the rat.

**Methods:**

Two different doses of tropisetron (5 and 10 mg/kg) or vehicle were administered intraperitoneally 30 min before pMCAO. Neurological deficit scores, mortality rate and infarct volume were determined 24 h after permanent focal cerebral ischemia.

**Results:**

Tropisetron failed to reduce cerebral infarction. Animals receiving tropisetron showed a significant increase (p < 0.05) in neurological deficits and mortality rate.

**Conclusion:**

Data from this study indicate that blockade of 5-HT_3 _receptors with tropisetron worsens ischemic brain injury induced by pMCAO. These findings could have important clinical implications. Patients taking tropisetron, and possibly other 5-HT_3 _antagonists, could potentially have a worse outcome following a brain infarct.

## Background

Stroke is a leading cause of death and disability worldwide [1,2]. Although significant efforts have been devoted to understand the pathophysiology of cerebral ischemia, very few therapeutic options are available [3]. Among the deleterious events following occlusion of a blood vessel in the brain, excitotoxicity and alteration of intraneuronal Ca^2+ ^homeostasis are considered early processes that propagate the cascade of harmful events leading to cerebral infarction [4,5]. The primary ischemic episode results in neuroinflammation, which leads to increase in tissue destruction through mechanisms involving oxidative stress, upregulation of pro-inflammatory cytokines (TNF-α, IL-1β), enhanced metabolism of arachidonic acid, breakdown of the blood-brain barrier, and edema [6-8].

Recently, there has been much interest in the potential neuroprotective efficacy of 5-hydroxytryptamine (5-HT) receptor subtype 3 (5-HT_3_) antagonists [9,10]. Blockade of 5-HT_3 _receptor with MDL72222 and Y-25130 reduced glutamate release, elevation of cytosolic Ca^2+ ^concentration, oxidative damage, and apoptotic neuronal cell death induced by either hydrogen peroxide or β-amyloid peptide [9,10]. Furthermore, an earlier study by Kagami-ishi and colleagues demonstrated that the 5-HT_3 _antagonist Y-25130 conferred protection against ischemia-induced decrease in CA1 field potential in rat hippocampal slices [11].

In the light of all this previous evidence, the present investigation was conducted to assess whether treatment with the 5-HT_3 _antagonist tropisetron would show neuroprotective effect against cerebral ischemic injury. Since most cases of human ischemic stroke are caused by irreversible occlusion of major cerebral arteries [12-14], we utilized a well-standardized model of permanent occlusion of the middle cerebral artery (pMCAO) in the rat.

## Results

### Effect of tropisetron on infarct volume following permanent focal cerebral ischemia in the rat

**Figure 1:**
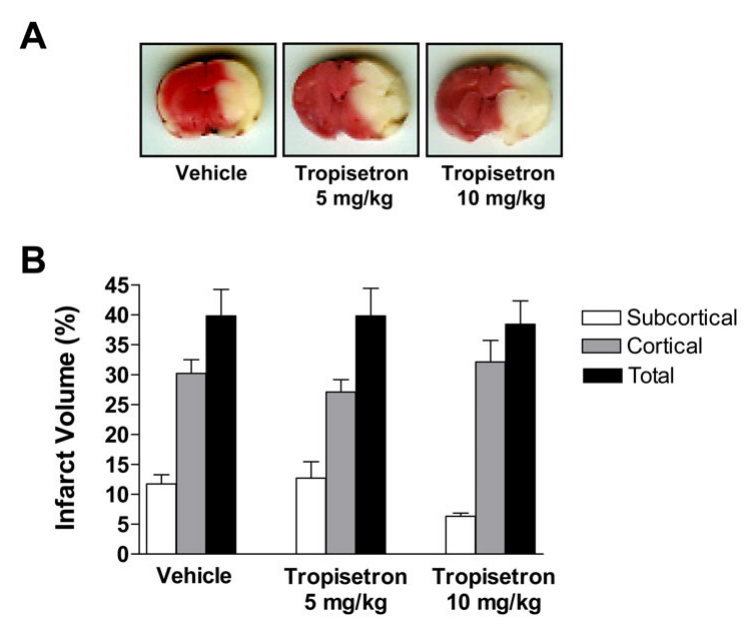
(**A**): **Representative TTC-stained sections of vehicle- and tropisetron-treated rats at 24 h following permanent middle cerebral artery occlusion (pMCAO).** Tropisetron or vehicle was given intraperitoneally 30 min before pMCAO. (**B**): Total, cortical and subcortical infarct volumes assessed at 24 h in control (vehicle) and tropisetron groups. No significant effect of tropisetron on cerebral infarction was found.

Using the intraluminal filament technique, we had previously found that infarct size reached its maximal values at 24 h after pMCAO [15]. For this reason, in the present investigation animals were studied at 24 h after the onset of stroke. All animals studied had large infarct areas in the MCA territory, involving both cortical and subcortical structures, as revealed by the lack of TTC staining in these regions (Fig. 1A). Pretreatment with two different doses of the 5-HT_3 _antagonist, tropisetron, failed to reduce the total infarct volume, as shown in Fig. 1B. To exclude the possibility of a region-specific outcome, we next investigated the effect of tropisetron on the infarct volume in cortical and subcortical regions. Again, tropisetron did not significantly modify the size of the infarct in this animal model of stroke (Fig. 1B). Similarly, we studied the rostrocaudal distribution of areas of cortical and subcortical infarction at six coronal levels in tropisetron- and vehicle-treated rats. No effect of tropisetron was observed at any level of the brain infarct (data not shown). Furthermore, among treatment groups, there were no statistically significant differences in the edema index (Vehicle: 1.033 ± 0.028; Tropisetron 5 mg/kg: 1.031 ± 0.013; and Tropisetron 10 mg/kg: 1.039 ± 0.018).

### Ischemic animals receiving tropisetron showed increased neurological deficits and mortality

**Figure 2:**
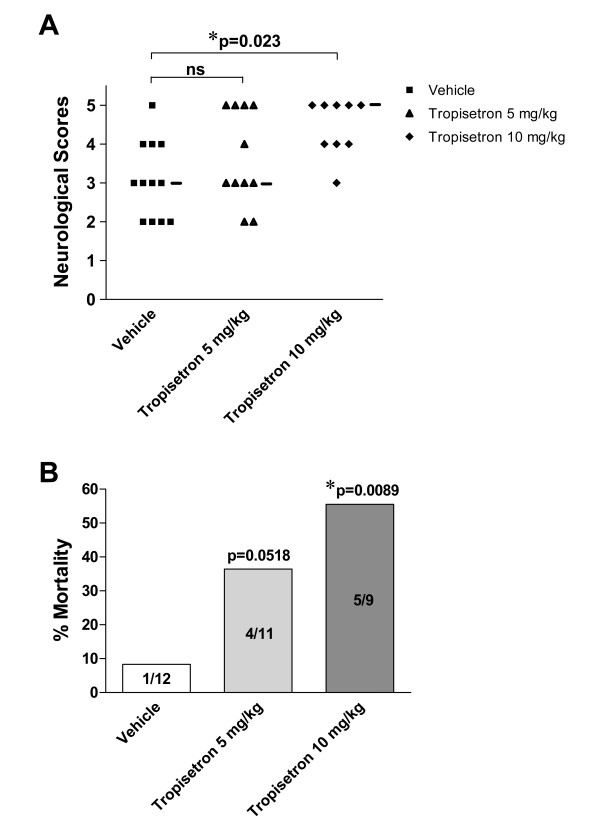
**Effect of the 5-HT_3 _antagonist tropisetron on neurological deficits scores **(**A**) **and mortality rate** (**B**) **after pMCAO.** Animals received a single i.p. injection of tropisetron or the vehicle 30 min before being subjected to pMCAO. Twenty-four hours later, neurological scores were evaluated following a 6-point scale as detailed in Materials and Methods. Median neurological score is marked in Panel A by a small horizontal bar. Mortality rate is presented as percentage (%). *p < 0.05 with respect to vehicle-treated animals.

Animals given tropisetron displayed a significant increase in the neurological deficits at 24 h after pMCAO. Although administration of 5 mg/kg of tropisetron did not significantly modify neurological scores, treatment of rats with 10 mg/kg of tropisetron 30 min before pMCAO produced a significant deterioration of the neurological status in these animals (Fig. 2A). Furthermore, in animals receiving the highest dose of tropisetron, there was a significant (p = 0.0089; Χ^2 ^test) increase to 55.5% (5 of 9 rats) in the mortality rate compared to vehicle-treated animals (8.3%; 1 of 12 rats), as shown in Fig. 2B. There was a trend towards an increase in mortality in rats receiving 5 mg/kg of tropisetron, but this effect did not reach statistical significance (p = 0.0518; Χ^2 ^test).

## Discussion

Present data do not support the notion that blockade of 5-HT_3 _receptors reduces ischemic brain injury induced by focal cerebral ischemia. On the contrary, our findings clearly showed that treatment with tropisetron worsened neurological recovery and increased mortality of ischemic animals. No previous investigations have been published on the effects of the 5-HT_3 _antagonist tropisetron on neuronal damage induced by focal cerebral ischemia. Thus, these *in vivo *data could have important implications for the potential use of 5-HT_3 _antagonists in neuroprotection, as other investigators have suggested [9-11]. Most 'negative' results in preclinical studies in cerebral ischemia are not even submitted for publication. This practice goes against the STAIR (Stroke Therapy Academic Industry Roundtable) recommendations that data, both positive and negative, should be published [16-18].

Previous studies evaluating the neuroprotective efficacy of 5-HT_3 _antagonists utilized *in vitro *cultures of neurons and hippocampal slices subjected to ischemia-like conditions. Those findings indicated that blockade of 5-HT_3 _receptors might confer significant neuroprotection by reducing glutamate release, free radicals formation, and maintenance of Ca^2+ ^homeostasis [9,10].

It is well established that the activation of 5-HT_3 _receptors is followed by rapid depolarization causing rapid rise in cytosolic Ca^2+ ^levels by inducing influx of Ca^2+ ^and mobilization of intracellular Ca^2+ ^stores [19]. These processes modulate the release of various neurotransmitters including dopamine, glutamate, acetylcholine, GABA and 5-HT itself [19,20].

We had previously shown that tropisetron potently inhibits lipopolysaccharide-mediated production of TNF-α and IL-1β in human monocytes, as well as 5-HT-induced prostaglandin E_2 _(PGE_2_) release from synovial cells [21,22]. In addition, we found that tropisetron inhibited DNA binding and transcriptional activity of NFAT, AP-1 and NF-κB in T cells exposed to pro-inflammatory stimuli [23]. Furthermore, very recent data from our lab indicate that the 5-HT_3 _antagonists, tropisetron, ondansetron and dolansetron, significantly reduce PGE_2 _release and 8-isoprostane formation (a reliable marker of free radical generation) in neuronal cells exposed to IL-1β (Fiebich et al., manuscript in preparation).

Based on this promising evidence coming from *in vitro *studies, one might expect that blockade of 5-HT_3 _receptors to be beneficial in cerebral ischemia. However, results from this investigation not only demonstrate that treatment with tropisetron failed to diminish brain infarction, but also showed that neurological deficits and mortality rate were dramatically increased in ischemic animals given this 5-HT_3 _antagonist. At present, the molecular mechanisms underlying these detrimental effects of tropisetron are far from clear. In addition, it is not yet known whether other 5-HT_3 _antagonists share these stroke-worsening properties of tropisetron.

One possible explanation for these detrimental effects of tropisetron might be the ability of 5-HT_3 _antagonists to significantly reduce GABAergic neurotransmission [24,25]. This notion is based on evidence showing that Ca^2+ ^influx through presynaptic 5-HT_3 _receptors facilitates GABA release in several brain regions [25,26]. Cerebral ischemia produces a dramatic increase in GABA release, which is thought to be an important compensatory mechanism against ischemia [27]. Activation of GABA receptors potently reduces ischemic and excitotoxic brain injury [27]. Thus, blockade of 5-HT_3 _receptors with tropisetron could potentially hamper this protective mechanism involving ischemia-mediated GABA release. However, this possibility should be confirmed experimentally in a model of ischemic stroke.

Antagonists of 5-HT_3 _receptors, including tropisetron, are clinically used for the management of postoperative nausea and vomiting (PONV), and for the treatment of chemotherapy-induced nausea and emesis. These drugs selectively and competitively bind to 5-HT3 receptors, blocking serotonin binding at vagal afferents in the gut and in the regions of the central nervous system involved in emesis, including the chemoreceptor trigger zone and the nucleus tractus solitarii [28,29].

It is difficult to ascertain whether the doses of tropisetron used here in the rat are comparable to the dosage used in humans. A direct interspecies dose extrapolation is usually inaccurate due to changes in the pharmacokinetic/pharmacological behavior of the drug in different species [30]. This is further complicated by the fact that some 5-HT_3 _antagonists, including tropisetron, show a bell-shaped dose-response curve [20], leaving open the possibility that lower doses of tropisetron might have complete different effects in permanent ischemic stroke.

Significant increase in neurological deficits and mortality was found in animals receiving tropisetron. However, no parallel increase in infarct size was observed in any of the groups treated with tropisetron when compared to rats given the vehicle. There is not always a direct correlation between the lesion size and the severity of neurological deficits as demonstrated before in animal models [31,32] and in stroke patients [33]. This indicates that the severity of disability is not predicted well by the amount of brain tissue lost.

## Conclusion

In summary, this study showed that the 5-HT_3 _antagonist tropisetron failed to reduce cerebral infarction induced by pMCAO in the rat, and produced a significant increase in neurological deficits and mortality rate. Whichever the mechanism(s) responsible for the detrimental effects of tropisetron in cerebral ischemia, present study could have important clinical implications. Patients taking tropisetron, and possibly other 5-HT_3 _antagonists, might have a worse prognosis following a cerebral ischemic event.

## Methods

### Animals

Male Sprague-Dawley rats weighing 280–320 g at the time of surgery were obtained from Harlan Spain (Harlan Interfauna Iberica S.A., Barcelona, Spain). Our institutional animal care and use committee approved the experimental protocol. The animals were quarantined for at least 7 days before the experiment. Animals were housed in groups in a room whose environment was maintained at 21–25°C, 45–50% humidity, and 12-h light/dark cycle. They had free access to pellet chow and water. Animal housing, care, and application of experimental procedures were in accordance with institutional guidelines under approved protocols.

### Induction of permanent middle cerebral artery occlusion (pMCAO) in the rat

Rats were anesthetized with chloral hydrate (300 mg/kg body weight, i.p.). Once surgical levels of anesthesia were attained (assessed by absence of hind leg withdrawal to pinch), ischemia was induced by using an occluding intraluminal suture as described previously [31,34,35]. Briefly, the right common carotid artery (CCA) was exposed by a ventral midline neck incision and ligated with a 3-0 silk suture. The pterygopalatine branch of the internal carotid artery was clipped to prevent incorrect insertion of the occluder filament. Arteriotomy was performed in the CCA approximately 3 mm proximal to the bifurcation and a silicone rubber coated 4-0 nylon filament (Doccol Corporation, Redlands, CA, USA) was introduced into the internal carotid artery (ICA) until a mild resistance was felt (18–19 mm). Mild resistance to this advancement indicated that the intraluminal occluder had entered the anterior cerebral artery and occluded the origin of the anterior cerebral artery, the middle cerebral artery (MCA) and posterior communicating arteries [36]. After the advancement of the nylon suture, the ICA was firmly ligated with a 3-0 silk suture. The incision was closed and the occluding suture was left in place until sacrificing the animals. The duration of surgery did not exceed 12 min in any case. The animals were allowed to recover from anesthesia and to eat and drink freely. The body temperature was strictly controlled during and after ischemia using an overhead lamp and a heating blanket. No significant effect of tropisetron on body temperature was observed (data not shown). To allow for better postoperative recovery, we chose not to monitor physiological parameters in the present study because additional surgical procedures are needed for this monitoring.

### Evaluation of neurological deficits

After 24 h of pMCAO, an unaware independent observer performed the neurological evaluations prior to the sacrifice of the animals according to a six-point scale: 0 = no neurological deficits, 1 = failure to extend left forepaw fully, 2 = circling to the left, 3 = falling to left, 4 = no spontaneous walking with a depressed level of consciousness, 5 = death [36,37].

### Quantification of brain infarct volume

The size of the brain infarct was assessed at 24 h following pMCAO. The method for quantification of infarct volume was performed exactly as described in these reports [15,35,38,39]. Briefly, the animals were sacrificed under deep anesthesia and brains were removed, frozen, and coronally sectioned into six 2-mm-thick slices (from rostral to caudal, first to sixth) using a rat brain matrix (World Precision Instruments, Sarasota, FL, USA). The brain slices were incubated for 30 min in a 2% solution of 2,3,5-triphenyltetrazolium chloride (TTC) (Sigma Chemical Co., Saint Louis, MO, USA) at 37°C and fixed by immersion in a 10% phosphate-buffered formalin solution. Six TTC-stained brain sections per animal were placed directly on the scanning screen of a color flatbed scanner (Hewlett Packard HP Scanjet 5370 C) within 7 days. Following image acquisition, the images were analyzed blindly using a commercial image processing software program (Photoshop, version 7.0, Adobe Systems; Mountain View, CA, USA). Measurements were made by manually outlining the margins of infarcted areas. The unstained area of the fixed brain section was defined as infarcted. Cortical and subcortical uncorrected infarcted areas and total hemispheric areas were calculated separately for each coronal slice. Total cortical and subcortical uncorrected infarct volumes were calculated by multiplying the infarcted area by the slice thickness and summing the volume of the six slices. A corrected infarct volume was calculated to compensate for the effect of brain edema. An edema index was calculated by dividing the total volume of the hemisphere ipsilateral to pMCAO by the total volume of the contralateral hemisphere. The actual infarct volume adjusted for edema was calculated by dividing the infarct volume by the edema index [40,41]. Infarct volumes are expressed as a percentage of the contralateral (control) hemisphere. The investigator who performed the image analysis was blinded to the study groups.

### Drug treatment

Animals were randomly allotted in three different groups: tropisetron 5 mg/kg (n = 11), tropisetron 10 mg/kg (n = 9) and vehicle (physiological saline, n = 12). Drugs were given intraperitoneally 30 min before inducing pMCAO. These doses were selected based on previous studies demonstrating effects of tropisetron in the central nervous system [42,43]. In addition, the pharmacokinetic profile of these doses of tropisetron has being well-characterized in the rat [44]. Tropisetron was obtained from the in-house pharmacy of the University of Freiburg Medical School (Freiburg, Germany).

### Data analysis

Data are presented as mean ± S.E.M. Values were compared using t-test (two groups) or one-way ANOVA with *post-hoc *Student-Newman-Keuls test (multiple comparison). Neurological deficit scores were analyzed by Kruskal-Wallis non-parametric ANOVA followed by the Dunn test (multiple comparison) or Mann-Whitney test for analysis of individual differences. Mortality rate was analyzed using Chi-square (Χ^2^) test. Differences were considered significant when p < 0.05.

## List of abbreviations

ANOVA (analysis of variance); 5-HT (5-hydroxytryptamine); pMCAO (permanent middle cerebral artery occlusion); TTC (2,3,5-triphenyltetrazolium chloride).

## Competing interests

The author(s) declare that they have no competing interests.

## Authors' contributions

ECJ designed and performed the study, reviewed the data, and wrote the manuscript. EM and BLF provided consultation, contributed to the design of the study, and reviewed the data. All authors read and approved the final manuscript.
